# Is Zinc Concentration in Toxic Phase Plasma Related to Dengue Severity and Level of Transaminases?

**DOI:** 10.1371/journal.pntd.0002287

**Published:** 2013-06-20

**Authors:** Kamolwish Laoprasopwattana, Chonthicha Tangcheewawatthanakul, Wanutsanun Tunyapanit, Rassamee Sangthong

**Affiliations:** 1 Department of Pediatrics, Faculty of Medicine, Prince of Songkla University, Hat Yai, Songkhla, Thailand; 2 Epidemiology Unit, Faculty of Medicine, Prince of Songkla University, Hat Yai, Songkhla, Thailand; London School of Hygiene and Tropical Medicine, United States of America

## Abstract

**Objective:**

To determine the relationship between plasma zinc values and the severity of dengue viral infection (DVI) and DVI-caused hepatitis.

**Methods:**

A prospective cohort study was conducted during 2008–2010 in hospitalized children aged <15 years confirmed with DVI. Complete blood count, aspartate aminotransferase (AST), alanine aminotransferase (ALT), and zinc values (mcg/dL) were determined twice: first during the toxic phase (Zn1) and secondly two weeks after recovery (Zn2).

**Results:**

39 patients were enrolled with a mean age of 9.7±3.7 years, and 15/39 diagnosed with dengue shock syndrome (DSS). Zn1 values were lower than Zn2 values [median (IQR): 46.0 (37.0, 58.0) vs 65.0 (58.0, 81.0) mcg/dL, respectively, p <0.01]. Zn1 but not Zn2 values had a negative correlation with AST and ALT (r_s_ = −0.33, p = 0.04 and r_s_ = −0.31, p = 0.05, respectively). Patients with DSS had lower Zn1 but not Zn2 values compared with non-DSS patients [median (IQR) Zn1, 38.0 (30.0, 48.0) vs 52.5 (41.2, 58.7), p = 0.02; Zn2, 61.0 (56.0, 88.0) vs 65.0 (59.5, 77.5), respectively, p = 0.76]. Zn1 values showed a decreasing trend across increasing dengue severity groups (p = 0.02). Age <5 years and DVI-associated diarrhea were associated with low Zn1.

**Conclusion:**

Children who had a higher grade of dengue disease severity and liver cell injury had lower Zn1 values. Low Zn1 values were probably caused by loss from diarrhea and from zinc translocating to liver cells.

## Author Summary

Dengue viral infection (DVI) is endemic in tropical counties and severe DVI is a significant cause of death, especially in children. Increased vascular endothelial permeability during the defervescence stage of DVI leading to plasma leakage plays an important role in dengue disease severity. Zinc is a protective and critical nutrient for maintenance of endothelial integrity, and also functions as an antioxidant and membrane stabilizer. Previous studies have found that zinc supplements in children who had diarrhea and sepsis improved the clinical outcomes. Zinc deficiency is common in school children, the age group that commonly acquires DVI, particularly in developing countries. However, prior to studying the potential benefits of zinc supplementation in DVI, having some baseline information concerning plasma zinc values and their correlation with dengue disease severity is necessary. We performed a prospective cohort study during 2008–2010 in 39 hospitalized children aged <15 years confirmed with DVI, and found that plasma zinc values during the toxic phase of disease showed a decreasing trend across increasing dengue severity groups, and also correlated with liver cell injury. DVI-associated diarrhea was probably a major cause of markedly decreased plasma zinc values. These findings will be useful as background information in further studies of whether zinc supplementation can improve the clinical outcome of DVI.

## Introduction

The immunopathogenesis of dengue viral infection (DVI) is not well understood, and the level of disease severity is multifactorial, depending on various factors such as viral virulence, secondary DVI, immune response to DVI, and host factors including genetic and nutritional status [1]–[3]. Plasma leakage during the toxic phase of illness, caused by increased endothelial permeability, plays an important role in dengue hemorrhagic fever (DHF)/dengue shock syndrome (DSS). Previous studies have found that dengue disease severity was associated with concentrations of pro-inflammatory cytokines and cell apoptosis [4]–[9].

Other studies have found that obese or malnourished children with DVI had higher morbidity and mortality rates than those with normal body weight, suggesting that nutritional status might play an important role in the immunopathogenesis of DVI [3], [4], and also that obese and malnourished children had higher proportions of zinc deficiency than normal body weight children [10]–[13]. Zinc deficiency is an important problem in school children, particularly in developing countries [13]. In Thailand, more than half of school children tested had zinc deficiency [14], [15].

Zinc also functions as an antioxidant and membrane stabilizer. Zinc deficiencies can result in inefficient clearing of infections by impairing innate and adaptive immune responses, creating an imbalance of pro- and anti-inflammatory cytokines, and induction of cell death via apoptosis [16]–[22].

Tumor necrotic factor (TNF) has been found to induce zinc deficient-endothelial cells to produce a higher number of inflammatory cytokines than non-zinc deficient endothelial cells, but the production of these inflammatory cytokines was partially inhibited by prior zinc supplementation, suggesting that zinc is a protective and critical nutrient for maintenance of endothelial integrity [23].

The liver is the major target organ of the dengue virus and severity of liver injury is associated with dengue disease severity. A previous study found that zinc supplementation in children who had chronic liver disease could prevent liver injury during treatment with pegylated interferon alpha and ribavirin [24].

These various findings suggest that zinc could play an important role in the immune response to DVI. To our knowledge, there has been only one study involving plasma zinc concentrations collected in the first day of admission in patients with DVI, which did not find any correlation between disease severity and zinc concentrations [25]. However, the findings of one study are not conclusive, as there could be factors that interfere with zinc concentrations at different stages of DVI, meaning the time of sample collection could be important. The current study collected plasma samples during both the toxic phase and then 2 weeks after the patient recovered in order to examine potential correlations between plasma zinc values and dengue disease severity and liver injury.

## Methods

A prospective cohort study was conducted in children <15 years of age who were hospitalized with DVI at Songklanagarind Hospital, Thailand, from January 2008 to August 2010. The severity of DVI was diagnosed according to the criteria of the World Health Organization (WHO) [26], primarily based on the presence of dengue IgM or a 4-fold increase in hemagglutination inhibition titers (HAI). Primary and secondary DVI were diagnosed if the HAI titers were <1∶1280 and ≥1∶2560, respectively. Dengue fever (DF) was diagnosed if the patient had acute febrile illness with or without hemorrhagic manifestation, and no evidence of plasma leakage. DHF was diagnosed if the patient fulfilled all of the following criteria: acute febrile illness, hemorrhagic manifestation, thrombocytopenia (<100,000 platelets/mm^3^), and evidence of plasma leakage as determined by hemoconcentration (hematocrit >20% above baseline), pleural or abdominal effusion (as revealed by radiography or another imaging method) or hypoalbuminemia. DHF grade I was diagnosed if the patient met all of the DHF criteria without evidence of circulatory failure. DHF grade II was diagnosed if the patient had evidence of a bleeding disorder. DHF grades III or IV (DSS) was diagnosed if the patient met all of the DHF criteria and there was also evidence of impending (narrow pulse pressure, <20 mmHg) or profound circulatory failure. Patients who had DF or DHF grades I or II were classified as non-DSS. Demographic characteristics and known potential risk factors for disease severity were recorded, including age, sex, weight standard deviation score (WSDS), obesity (WSDS >2), underweight (WSDS <−2), and severity of DVI according to the WHO criteria [26]. Hepatic failure was defined by the rapid development of severe acute liver injury with impaired synthetic function and encephalopathy in a patient with no history of liver disease. Dual infection was defined as a second non-DVI detected within 3 days after admission to the hospital.

To determine the severity of plasma leakage and hepatitis, the highest and lowest values of hematocrit and liver function tests (LFTs), including aspartate aminotransferase (AST), alanine aminotransferase (ALT), total bilirubin, direct bilirubin, albumin, and alkaline phosphatase (ALP), were measured. Plasma zinc values, complete blood counts (CBCs), and LFTs were determined twice, first during the toxic phase (within 24 hours after defervescence or shock and before receiving blood products or colloidal fluid), and secondly 2 weeks after recovery. The plasma zinc levels were measured by flame-atomic absorption spectrophotometer (Varian Techtron, Australia) [27]. The normal plasma zinc level in a healthy child is 70–120 mcg/dL, and moderately and markedly decreased plasma zinc\were deemed in our patients at 40–60 and <40 mcg/dL, respectively.

### Ethics statement

Permission from the institutional review board of Prince of Songkla University was obtained prior to conducting the study. Parents/guardians provided written, informed consent on behalf of all child participants.

### Statistical analysis

Descriptive statistics were used to describe the baseline characteristics of the patients. Comparisons of variables between patients with and without DSS, and with and without severe zinc deficiency, were made using the Mann-Whitney U-test. Fisher's exact test was used for comparison of categorical variables.

Zinc levels in the toxic phase were compared graphically with those in the recovery phase and compared statistically using the Wilcoxon -signed rank test and Spearman correlation coefficient. Zinc levels in the toxic phase and in recovery phase were compared across dengue severity groups using a non-parametric test for trends [28]. The correlation of zinc with the AST and ALT levels in the toxic phase were examined using Spearman correlation.

A p-value of <0.05 was considered statistically significant. All analyses were performed using Stata version 10 (StataCorp, College Station, Texas).

## Results

### Clinical characteristics

Of the 39 patients admitted during the study period with DVI, 22 (56.4%) were male and the mean age was 9.7±3.7 years (range 9 months to 14 years). Of these, 6/39 (15.4%) were obese and none were underweight. The median WSDS was 0.2 (range −1.9 to 4.1). DF and DHF grades I, II, III, and IV were diagnosed in 7, 12, 5, 13, and 2 patients, respectively. Primary and secondary DVI were diagnosed in 3 and 36 patients, respectively. Of the 3 patients who had primary DVI, 2 had DHF grade III (both were infants, aged 9 months and 1 year), and the other had DHF grade II (age 7.3 years).

Nausea or vomiting, upper respiratory symptoms (cough or runny nose) and diarrhea were found in 84.6%, 20.5%, and 23.1% of the cases, respectively. Dual infections were found in 3 patients with DSS, one each of urinary tract infection, shigellosis, and scrotal cellulitis. All 3 patients who had dual infection also had diarrhea.

None of the patients in the study died from the disease. Five of the DSS patients developed hepatic encephalopathy, while the patient with DHF grade IV with hepatic failure also had respiratory failure and active bleeding. Of the 16 patients who had hemorrhagic symptoms, 4 needed a blood transfusion to control bleeding.

### Plasma zinc values

**Figure 1:**
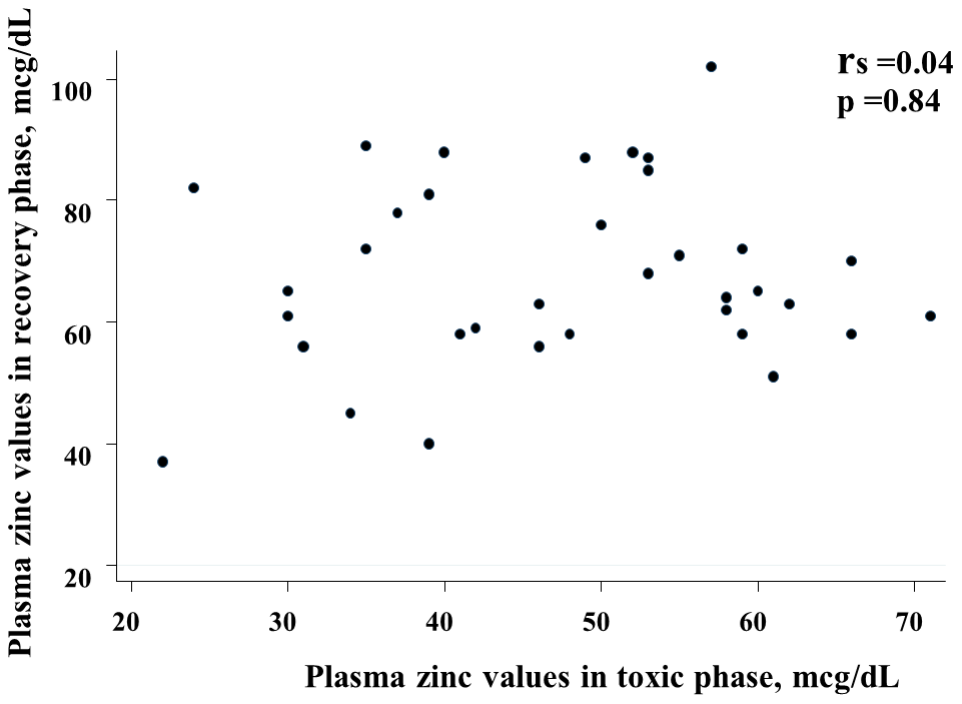
The relationship between plasma zinc levels during toxic and recovery phases.

The parent/guardian of the four patients did not allow their blood to be sampled during the recovery phase and are therefore omitted from the comparison with blood parameters in the toxic phase. In the remaining patients, the plasma zinc values measured from samples taken during the toxic phase were significantly lower than in blood collected 2 weeks after recovery [median (IQR): 46.0 (37.0, 58.0) vs 65.0 (58.0, 81.0) mcg/dL, respectively, p<0.01]. There was no correlation between plasma zinc values collected during the toxic phase and after recovery (r_s_ = 0.04; p = 0.84) (Figure 1).

During the toxic phase, all 39 patients except one had zinc values less than 70 mcg/dL; moderately (40–60 mcg/dL) and markedly decreased plasma zinc values (<40 mcg/dL) were found in 21 (53.8%) and 13 (33.3%) patients, respectively. All 3 patients with a dual bacterial infection, all 5 patients with hepatic encephalopathy, and 7/9 patients with acute diarrhea had plasma zinc values <40 mcg/dL. The duration of admission was longer in those who had zinc values lower than 40 mcg/dL than in those who had zinc values higher than 40 mcg/dL [median (IQR): 4 (3, 8) vs 3 (2, 4) days, p<0.01]. The proportions of gender, or patients with respiratory symptoms, nausea or vomiting, obesity, or hemorrhagic symptoms were not different in those who had plasma zinc values lower or higher than 40 mcg/dL. The median plasma zinc values were lower in those younger than 5 years, having diarrhea, dual infection, DSS, or hepatic encephalopathy compared to those who did not have these conditions (Table 1).

**Table 1:** Toxic phase plasma zinc values in relation to different factors.

Factor	Zinc level (mcg/dL), median (IQR), (n)	P*
Age ≤5 years		<0.01
Yes	31.0 (23.0, 37.0) (5)	
No	49.5 (39.7, 58.2) (34)	
Obese		0.83
Yes	46.5 (36.7, 59.5) (6)	
No	46.0 (36.0, 57.5) (33)	
Nausea or vomiting		0.85
Yes	46.0 (37.5, 57.5) (33)	
No	49.5 (33.7, 59.2) (6)	
Diarrhea		<0.01
Yes	35.0 (27.5, 40.0) (9)	
No	51.0 (40.7, 59.0) (30)	
Dual bacterial infection		0.02
Yes	30.0 (24.0, 35.0) (3)	
No	48.5 (39.0, 58.0) (36)	
Dengue shock syndrome		0.02
Yes	38.0 (38.0, 48.0) (15)	
No	52.5 (41.2, 58.7) (24)	
Hepatic encephalopathy		<0.01
Yes	30.0 (23.0, 33.0) (5)	
No	49.5 (39.7, 58.2) (34)	
Hemorrhagic symptoms		0.23
Yes	39.5 (30.2, 57.7) (16)	
No	48.0 (40.0, 58.0) (23)	

* Mann-Whitney U-test.

**Figure 2:**
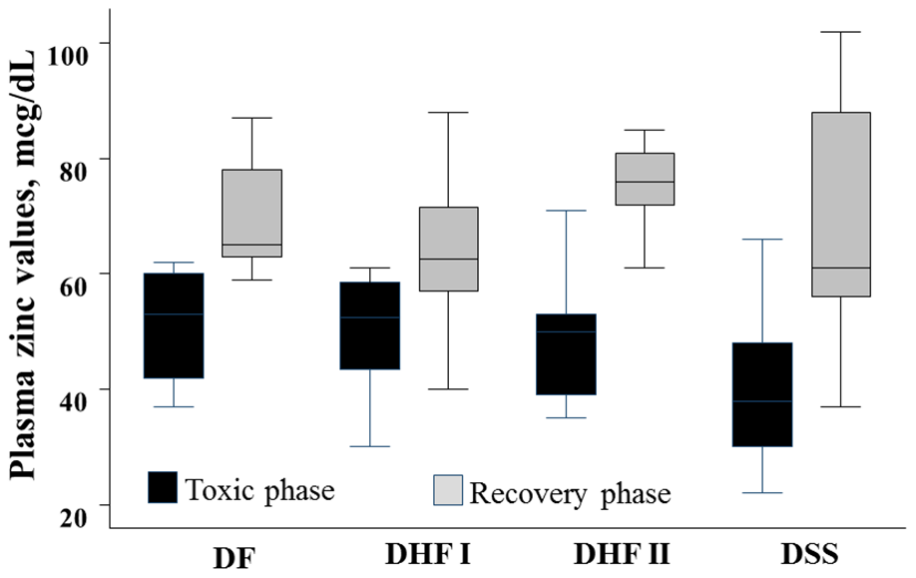
Plasma zinc concentrations during toxic and recovery phases according to disease severity.

The zinc value in the toxic phase showed a significant decreasing trend across increasing dengue disease severity groups in which the median (IQR) plasma zinc values in patients with DF, DHF grades I and II, and DSS were 53.0 (42.0, 60.0), 52.5 (42.2, 58.7), 50.0 (37.0, 62.0) and 38.0 (30.0, 48.0) mcg/dL, respectively, p = 0.02 (Figure 2). When the patients were classified into DSS and non-DSS groups, the plasma zinc values in the DSS group were significantly lower than in the non-DSS group [median (IQR): 38.0 (30.0, 48.0) vs 52.5 (41.2, 58.7) mcg/dL, respectively, p = 0.02].

Two weeks after recovery, the average plasma zinc values had increased in all dengue severity groups. Of the 35 patients who had a blood test in the recovery phase, 20 (57.1%) had zinc values lower than 70 mcg/dL. The plasma zinc values in patients with DF (n = 7) and DHF grades I (n = 12), II (n = 5), DSS (n = 11) were not significantly different, at medians (IQR) 65.0 (63.0, 78.0), 62.5 (56.5, 71.5), 76.0 (66.50, 83.0) and 61.0 (56.0, 88.0) mcg/dL, respectively, p = 0.97 (Figure 2). Plasma zinc values in those with and without DSS were not significantly different, at medians (IQR) 61.0 (56.0, 88.0) vs 65.0 (59.5, 77.5), respectively, p = 0.76. Plasma zinc values measured during this period were not correlated with disease severity. Obese and non-obese patients had zinc values <70 mcg/dL in 4/5 (80.0%) patients and 17/30 (56.7%) patients, respectively. The median (IQR) plasma zinc values in obese and non-obese patients were 58.0 (45.5, 68.5) and 66.5 (58.7, 82.7) mcg/dL, respectively, p = 0.07.

### Correlation between plasma zinc values and liver function and complete blood count

**Figure 3:**
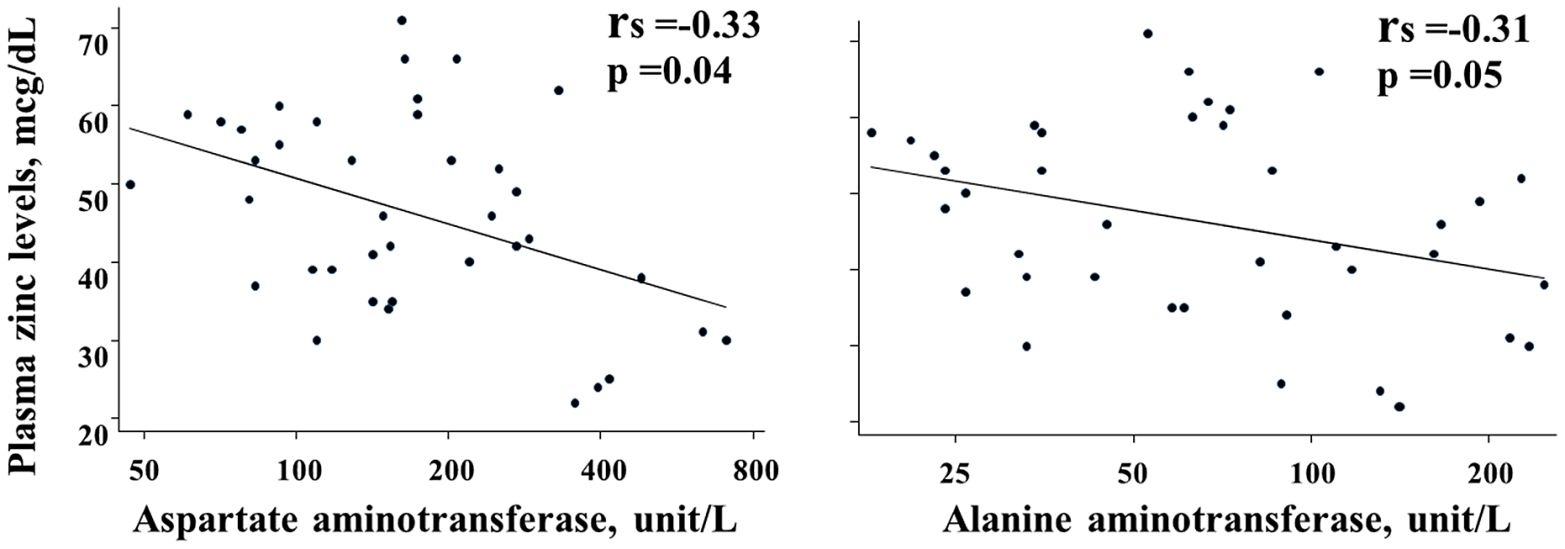
Plasma zinc concentrations had negative correlations with aspartate and alanine aminotransferase values.

During the toxic phase, plasma zinc values had a reverse correlation with both AST and ALT (r_s_ = −0.33, p = 0.04 and r_s_ = −0.31, p = 0.05, respectively) (Figure 3). However, the plasma zinc levels had no correlation with albumin and ALP levels, or with total numbers of white blood cells, lymphocytes, or platelets.

Two weeks after recovery, all of the LFTs and CBCs had returned to normal values, and the plasma zinc values had no correlation with AST, ALT, albumin, or ALP values, or total numbers of white blood cells, lymphocytes, or platelets.

## Discussion

In this study, most of our patients with DVI had low plasma zinc values when measured during the toxic phase of illness. Patients with DSS had lower plasma zinc concentrations than non-DSS patients, and plasma zinc concentrations had a negative correlation with liver enzymes. Plasma zinc concentrations collected 2 weeks after discharge from the hospital had returned to normal values in half of the patients.

The only other study we know of that has examined zinc levels in DVI patients was a study by Widagdo in 2008. Widagdo found low plasma zinc values (≤60 mcg/dL) in 34/45 (75.6%) children with DVI; the plasma zinc values were lowest in patients with DHF grade IV (plasma zinc values in DHF grades I, II, III and IV were 39.9±43.2, 45.1±41.2, 80.4±31.4, and 7.8±1.3 mcg/dL, respectively) [25]. We too found low plasma zinc values (≤60 mcg/dL) in most of our cases (87.2%). However, unlike our study which found that zinc levels in the toxic phase showed a significant decreasing trend across increasing dengue disease severity, the Widagdo study found a discordance between plasma zinc values and disease severity, in which patients with DHF grade III had higher plasma zinc values than those with DHF grades I and II. The Widagdo study did not find any correlations between plasma zinc values and diarrhea as in our study, but found a negative correlation between plasma zinc value and lymphocyte count, which we did not find. These different results between our study and Widagdo could be explained by noting the different times when blood samples were collected for plasma zinc assays; in the Widagdo study, the samples were collected on the first day of admission, but in our study we collected the blood during the toxic phase of illness when the inflammatory cytokines were surging, which is the period during which zinc homeostasis is most likely to be affected during DVI [4]–[9].

We found DVI-associated diarrhea in 9/39 (23.1%) patients, which was similar to previous studies which found rates of DVI-associated diarrhea of 17–35% [29], [30]. One study in adult patients found that patients who had DVI-associated diarrhea were more likely to have more severe DVI compared to those who had no diarrhea [31]. Although we found a higher proportion of DVI-associated diarrhea in patients with DSS vs non-DSS patients (33.3% vs 16.7%, respectively), the difference was not significant. DVI-associated diarrhea might be explained by an increased number of inflammatory cytokines, which directly affects and leads to zinc loss through the gastrointestinal tract [32], which we found to be the most likely factor explaining the decreasing plasma zinc values during the toxic phase of DVI. We found, as in the Widagdo study, that plasma zinc values during DVI did not correlate with nutritional status [25]. We also found that plasma zinc values during the toxic phase did not correlate with poor appetite during illness, but they did correlate with disease severity and liver injury. In addition, 2 weeks after recovery, the average plasma zinc values had markedly increased by 1.4 times the plasma values during the toxic phase. These findings suggest that during the toxic phase of DVI, inflammatory response and liver injury cause zinc translocation from the plasma into the liver to prevent oxidative damage to liver tissue, and then after recovery, the zinc translocates again, this time from the liver to the plasma, causing post-illness plasma zinc values to markedly increase compared to toxic phase plasma values [33], .

AST and ALT values in this study had only a moderate negative correlation with zinc concentrations, and therefore there are obviously other important factors accounting for lowered zinc levels during the toxic phase of DVI. Diarrhea appears to be one such factor influencing decreased plasma zinc levels.

Although 2 weeks after recovery all of our patients had a normal appetite, and the clinical profiles of illness and LFTs had returned to normal, the plasma zinc values of only half of the patients had returned to normal values (≥70 mcg/dL). When taken in light of previous studies in Thailand which found that more than half of school children tested had zinc deficiency [14], [15], these findings suggest that our patients' baseline plasma zinc values may have been low to begin with, and the most likely cause of zinc deficiency would be a low-zinc diet. Although our study found that obese children had lower zinc values than those who were not obese, there were too few obese patients in the study to attach any significance to this finding. We did not find any correlation of post-illness plasma zinc values and dengue disease severity and liver injury. We also note that plasma zinc values measured 2 weeks after the patients recovered cannot be assumed to represent the pre-illness plasma zinc values and thus speculate about whether pre-illness zinc status may be associated with dengue disease severity. To explore this question, researchers would have to know the pre-illness plasma zinc status of enrolled patients, which would rather impractically involve monitoring the plasma zinc values of a large number of children, on the chance that some of them would later develop DVI.

Although in normal humans 75% of the plasma zinc is loosely bound to albumin, and zinc is a component of ALP [35], we did not find any correlation between values of plasma zinc and albumin or ALP in samples collected either during the toxic phase or 2 weeks after recovery.

Taking these factors together suggests that the study patients who had a high inflammatory cytokines response to DVI could have subsequently developed severe DVI, liver injury, and low plasma zinc values from zinc loss, partially from diarrhea and partially from zinc translocating to liver cells.

Although our findings are potentially important in considering modifications to current DVI management, we do note that we had a small sample size, especially in regards to the number of patients with DHF grade IV, and future studies with a sufficient sample size are required to further explore our findings and tentative conclusions.

## Supporting Information

Checklist S1 STROBE checklist.
(DOC)
